# Research progress on the application of functional magnetic resonance imaging in cognitive dysfunction in patients with cerebral small vessel disease

**DOI:** 10.3389/fneur.2025.1622274

**Published:** 2025-08-18

**Authors:** Yingying Cao, Zhenzhen Lai, Jingwen Wang, Bijun Ye, Weiqiang Fan, Jiajia Ruan

**Affiliations:** Department of Neurology, Tiantai People's Hospital of Zhejiang Province (Tiantai Branch of Zhejiang Provincial People's Hospital), Hangzhou Medical College, Taizhou, China

**Keywords:** cerebral small vessel disease, functional magnetic resonance imaging, cognitive dysfunction, multimodal neuroimaging, biomarkers

## Abstract

Cerebral small vessel disease (CSVD) has recently garnered extensive attention owing to its significant disease burden, insidious onset, and the absence of effective specific treatments. Poor lifestyle habits and chronic diseases are closely linked to its occurrence and development, eventually resulting in cognitive dysfunction. Therefore, improvement of lifestyle, stable blood pressure, effective glucose lowering, low-salt and low-fat diet, smoking cessation, moderate exercise and adequate sleep are the keys to preventing cognitive dysfunction in cerebral small-vessel disease. Early prevention and intervention are of significant clinical importance and social value, particularly as CSVD represents a major contributor to cognitive dysfunction in approximately 40 million elderly individuals worldwide. This comprehensive review integrates findings across four functional MRI techniques—diffusion tensor imaging (DTI), resting-state functional MRI (rs-fMRI), magnetic resonance spectroscopy (MRS), and arterial spin labeling (ASL)—to provide a holistic framework connecting structural abnormalities with functional deficits in CSVD. This paper aimed to cover four aspects: an overview of CSVD, the correlation between the clinical manifestations of CSVD and cognitive dysfunction, the neuroradiological features of CSVD, and the application of functional magnetic resonance imaging (fMRI) in CSVD patients with cognitive dysfunction. The integration of these complementary techniques offers unprecedented insights into disease mechanisms, enabling improved early diagnosis, establishment of reliable imaging biomarkers for monitoring disease progression, and development of tailored therapeutic strategies to slow or prevent cognitive decline in affected individuals.

## Graphical abstract

**Figure:**
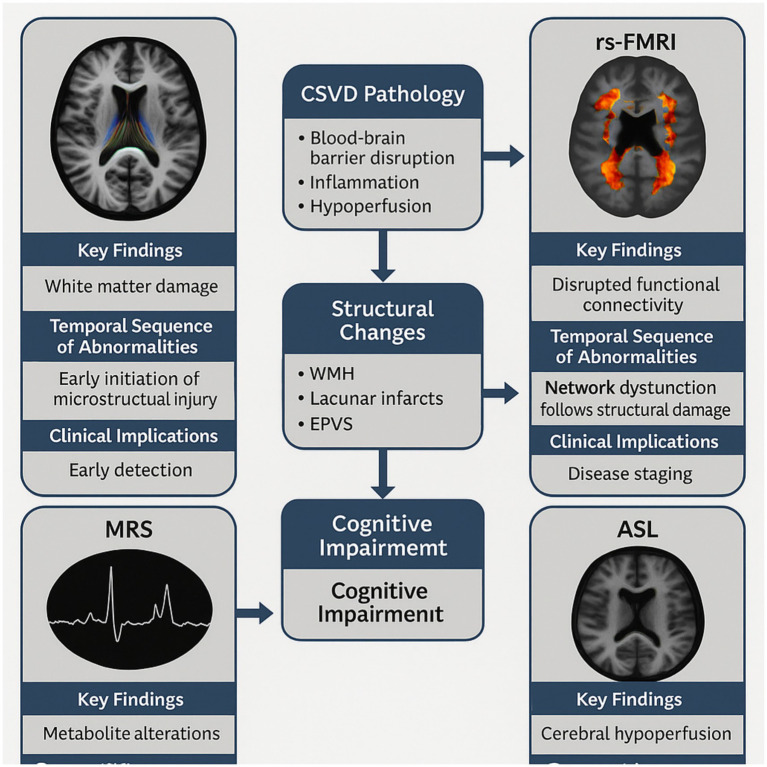
Multimodal Functional MRI Framework in CSVD-Related Cognitive Dysfunction. This graphical abstract illustrates the comprehensive framework of functional MRI applications in CSVD. The central panel shows the progression from CSVD pathology (involving blood–brain barrier disruption, inflammation, and hypoperfusion) to structural changes (WMH, CMBs, lacunar infarcts, EPVS), leading to network dysfunction and ultimately cognitive impairment. The surrounding panels depict the four functional MRI techniques (DTI, rs-fMRI, MRS, and ASL) with their key findings, temporal sequence of abnormalities, and clinical implications. The integration of these techniques enables early detection, disease staging, and personalized interventions.

## Introduction

With advances in modern medicine and an aging population, the increased lifespan has led to a rise in the number of elderly people suffering from cognitive dysfunction. At present, approximately 40 million elderly individuals worldwide are affected by cognitive dysfunction, with CSVD being a major contributor to cognitive dysfunction in this population, especially prevalent among those aged 60 years and above (1). As is well documented, its impact on cognitive function largely stems from small vessel pathology leading to medial temporal lobe atrophy, lacunar infarctions, and white matter damage. Pathologically, it is characterized by cerebral arteriosclerosis, fibrinoid necrosis, lipohyalinosis, amyloid angiopathy, and thrombosis in veins and venous sinuses, thereby eliciting cognitive, balance, and emotional disturbances (2). Meanwhile, distinctive radiological features encompass cerebral microbleeds (CMBs), lacunar infarctions (LI), perivascular spaces (PVS), cerebral atrophy, white matter lesions (WML), and recent small subcortical infarcts (RSSI) (1). Patients with CSVD have a rapid disease progression, so minimizing disease progression and intervening early are essential for the prevention and treatment of CVSD.

## Methodology

For this comprehensive systematic review, we conducted a thorough search of the literature published between January 2000 and January 2024 using the following electronic databases: PubMed, Web of Science, Scopus, and EMBASE. We employed a structured search strategy using the terms “cerebral small vessel disease” OR “CSVD” OR “white matter hyperintensities” OR “cerebral microbleeds” OR “lacunar infarct” AND “cognitive” OR “cognition” OR “cognitive impairment” OR “cognitive dysfunction” OR “dementia” AND “functional MRI” OR “fMRI” OR “diffusion tensor imaging” OR “DTI” OR “arterial spin labeling” OR “ASL” OR “magnetic resonance spectroscopy” OR “MRS.”

Inclusion criteria were: (1) original research or review articles focusing on CSVD and cognitive function; (2) studies employing at least one functional MRI technique; (3) human studies; (4) articles published in English. Exclusion criteria were: (1) case reports; (2) conference abstracts; (3) studies without cognitive assessments; (4) studies exclusively focused on other cerebrovascular diseases without specific CSVD assessment.

**Figure 1:**
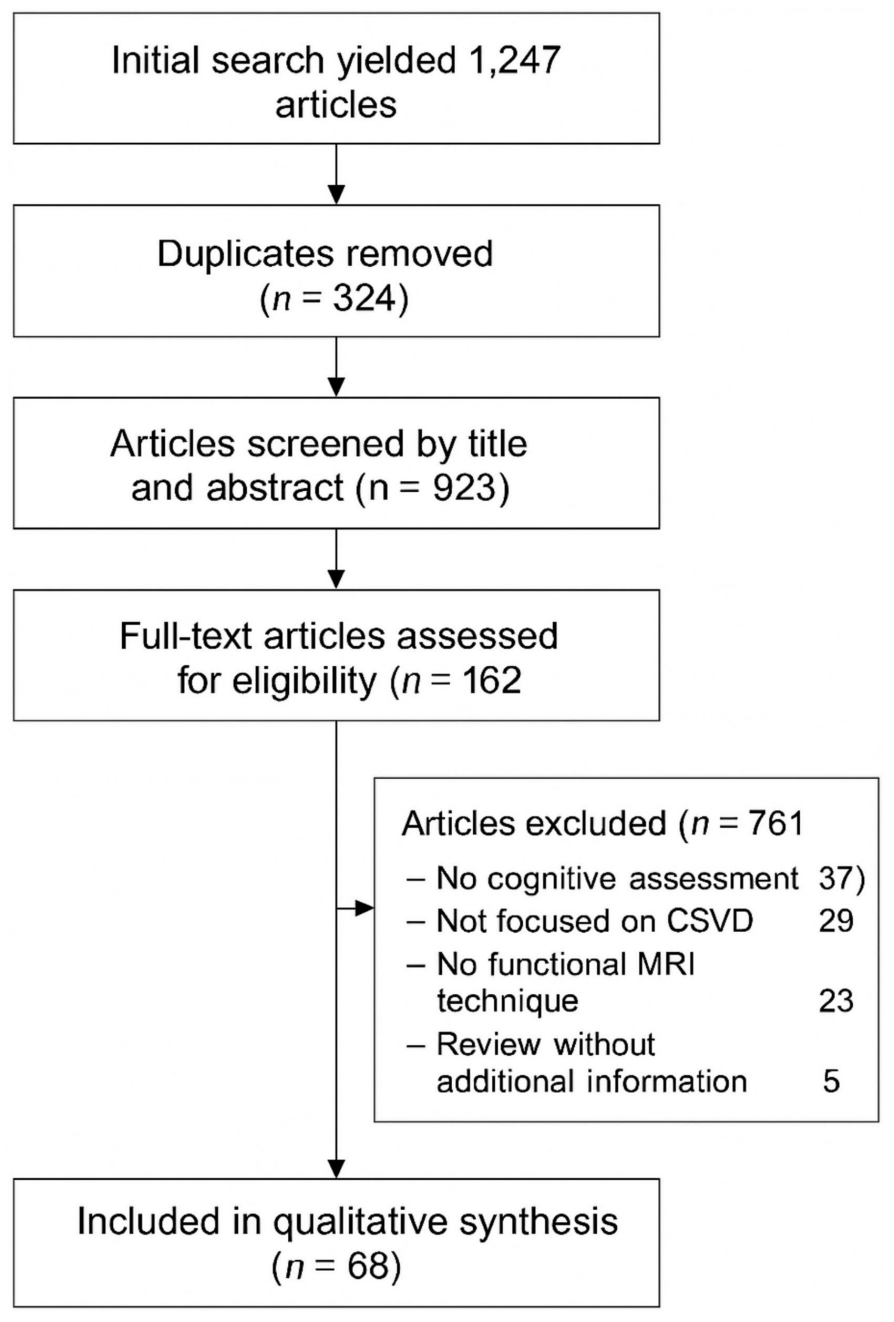
PRISMA flow diagram for study selection. PRISMA flow diagram illustrating the study selection process. Of 1,247 records identified through database searching, 923 remained after duplicate removal. After screening titles and abstracts, 162 full-text articles were assessed for eligibility. Finally, 68 studies were included in the qualitative synthesis after excluding 94 articles for reasons including: no cognitive assessment (*n* = 37), not focused on CSVD (*n* = 29), no functional MRI technique (*n* = 23), and review without additional information (*n* = 5).

Initial search yielded 1,247 articles. After removing duplicates (*n* = 324), 923 articles were screened by title and abstract, resulting in exclusion of 761 articles. The remaining 162 articles underwent full-text review, with 94 articles excluded for various reasons (no cognitive assessment, *n* = 37; not focused on CSVD, *n* = 29; no functional MRI technique, *n* = 23; review without additional information, *n* = 5). A total of 68 articles were included in the final qualitative synthesis. The quality of included studies was assessed using the Newcastle-Ottawa Scale for observational studies and AMSTAR-2 for systematic reviews (see Figure 1).

### Overview of cerebral small vessel disease

**Figure 2:**
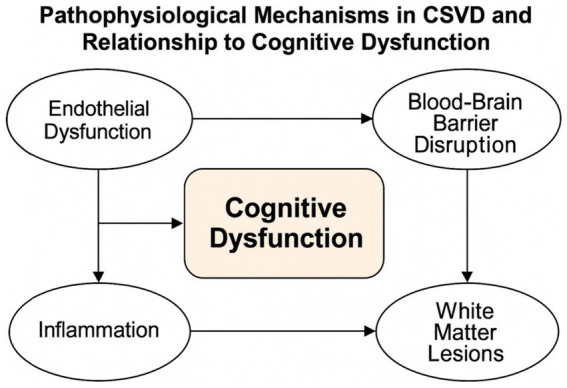
Pathophysiological mechanisms in CSVD and relationship to cognitive dysfunction. A schematic illustration of the pathophysiological cascade in CSVD leading to cognitive dysfunction. The progression begins with initial risk factors (hypertension, aging, diabetes, etc.) that trigger primary pathophysiological mechanisms (endothelial dysfunction, blood–brain barrier disruption, chronic hypoperfusion, inflammation, and oxidative stress). These mechanisms lead to secondary tissue damage (white matter lesions, lacunar infarcts, and cerebral microbleeds), which disrupts structural and functional brain networks, ultimately resulting in cognitive dysfunction.

Cerebral small vessel disease (CSVD) involves small cerebral vessels with diameters ranging from 30 to 800 μm, such as small intracerebral arteries, venules, and capillaries. It presents as a combination of clinical, imaging, and pathological syndromes, predominantly manifesting as strokes, cognitive decline, depression, gait disorders, and urinary dysfunction. Attributed to the small size and the numerous affected vessels, as well as the lack of specificity and difficulties in collecting biopsy tissues, it is challenging to accurately diagnose CSVD via clinical pathology. Presently, diagnosis primarily relies on imaging characteristics, with Magnetic Resonance Imaging (MRI) gaining considerable importance. Recent advancements in functional MRI technology have expanded our understanding of the relationship between CSVD and cognitive dysfunction by shifting from solely imaging-based detection to functional cognitive assessments. Early detection and prevention of CSVD are crucial due to its insidious onset and individual course, significantly alleviating social and personal stress (see Figure 2).

Pathologically, CSVD can be categorized into arteriosclerotic small vessel disease, sporadic or hereditary cerebral amyloid angiopathy, inflammatory and immune-mediated small vessel disease, venous collagenosis, and hereditary small vessel diseases distinct from cerebral amyloid angiopathy. Recent studies suggest that the pathophysiological mechanisms of CSVD involve a complex, interrelated cascade of events. We propose a systematic framework of these mechanisms, where each process influences and amplifies others:

1. Endothelial dysfunction: This represents the initial step in CSVD pathogenesis. Studies by Poggesi et al. (98) have established a chronic ischemic CSVD experimental model demonstrating that endothelial cell damage triggers a cascade of events affecting vessel integrity. The damaged endothelium produces reduced levels of nitric oxide and increased endothelin-1, leading to impaired cerebrovascular reactivity and autoregulation (3).
2. Blood-cerebrospinal fluid barrier disruption: Endothelial dysfunction directly contributes to increased permeability of the blood–brain barrier. Stamatovic et al. (3) have shown that this disruption allows plasma proteins and inflammatory mediators to enter the perivascular space and brain parenchyma, exacerbating tissue damage. Recent work has also identified decreased expression of tight junction proteins (claudin-5, occludin, ZO-1) in CSVD models, further compromising barrier integrity.
3. Chronic ischemia/hypoperfusion: The combined effects of endothelial dysfunction and barrier disruption impair small vessel autoregulation, leading to reduced cerebral blood flow. This chronic hypoperfusion creates an environment of persistent mild ischemia in deep white matter regions, which are particularly vulnerable due to their watershed vascular supply.
4. Inflammatory response: While previously underappreciated in CSVD pathogenesis, inflammation plays a crucial role in both initiating and perpetuating tissue damage. Recent studies have identified elevated levels of pro-inflammatory cytokines (IL-1β, IL-6, TNF-*α*) in the cerebrospinal fluid and blood of CSVD patients. Inflammation contributes to CSVD through multiple mechanisms: (a) Microglial activation, demonstrated by increased expression of microglial markers (Iba-1, CD68) in CSVD-affected white matter (b) Astrogliosis, characterized by reactive astrocytes that produce inflammatory mediators (c) Complement system activation, which enhances inflammatory damage (d) Perivascular inflammation, which directly damages small vessel walls (e) Blood–brain barrier disruption, creating a vicious cycle of increasing permeability.
5. Oxidative stress: Chronic ischemia and inflammation generate excessive reactive oxygen species (ROS) that overwhelm antioxidant defenses. Oxidative stress directly damages cellular components including lipids, proteins, and DNA in both vessels and neurons, contributing to cellular dysfunction and death.
6. Small vessel wall alterations: The cumulative effects of these processes lead to structural changes in small vessel walls, including fibrinoid necrosis, lipohyalinosis, and hyaline arteriolosclerosis. These changes further compromise vessel function and can lead to vessel occlusion or microhemorrhages.
7. Neuronal injury and oligodendrocyte damage: The end result of these cascading processes is damage to neuronal circuits and myelin-producing oligodendrocytes, manifesting as the characteristic white matter hyperintensities, lacunar infarcts, and microbleeds seen on imaging.

This integrated pathophysiological framework helps explain the progressive nature of CSVD and its widespread effects on brain structure and function, ultimately leading to cognitive impairment. Recent studies suggest that its pathophysiological mechanisms may involve endothelial injury, chronic ischemia/hypoxia, blood-cerebrospinal fluid barrier (BCFB) impairment, and inflammation, with BCFB disruption and endothelial injury playing key roles. Poggesi et al. (98) established a chronic ischemic CSVD experimental model to examine blood-cerebrospinal fluid barrier damage. At the same time, other studies indicated that endothelial dysfunction could increase the permeability of the blood-cerebrospinal fluid barrier, further exacerbating brain parenchymal damage (3). Thus, BCFB disruption and endothelial injury can impair small vessel autoregulation, leading to reduced cerebral blood flow and chronic cerebral hypoperfusion, ultimately manifesting as white matter lesions, occlusion of small vessels causing ischemic necrosis, and vessel wall injury leading to cerebral microbleeds in neuroimaging.

Among the risk factors associated with CSVD, hypertension and age are considered the most definitive (4). Long-term hypertension can drive arteriosclerosis and hyalinosis, leading to narrowing or occlusion of small vessels, further increasing the risk of CSVD. A cross-sectional study demonstrated that hypertension can alter the integrity of white matter structure, with blood pressure levels linearly correlating with overall white matter structure (5). With aging, the detection rates of CSVD and cognitive dysfunction increase (6). Thompson and Haki (99) proposed that old age can cause global small vessel dysfunction, leading to CSVD. The number of patients with hyperlipidemia and diabetes has been increasing in recent years, and both have been confirmed as significant risk factors for CSVD. The study undertaken by Lee et al. (7) documented that long-term consumption of high-fat foods, especially those rich in saturated fats, increases the permeability of the blood–brain barrier, thereby aggravating cognitive dysfunction. Diabetes increases the risk of CSVD, leading to cognitive dysfunction and dementia through disturbed glucose metabolism and chronic hyperglycemia, consequently promoting anaerobic metabolism and acidosis and eventually leading to neuroinflammation (7). Another significant and independent risk factor for CSVD is serum homocysteine, which can directly damage the vascular endothelium, modulate platelet activity, affect the oxidation of low-density lipoproteins, and accelerate thrombosis and arteriosclerosis (8). Zhang et al. (9) noted that the degree of cognitive dysfunction in patients was positively correlated with serum homocysteine levels, with a sensitivity of 75.61% for diagnosing CSVD with cognitive dysfunction. Additionally, stroke, alcohol abuse, smoking, or a history of transient cerebral ischemia are also significant risk factors for CSVD.

### Clinical manifestations of cerebral small vessel disease and its correlation with cognitive dysfunction

Cerebral small vessel disease (CSVD) is a significant cause of cerebral microbleeds and lacunar strokes, with 42.3% of ischemic strokes being lacunar in nature. Lacunar infarctions chiefly manifest as pure motor strokes, pure sensory strokes, ataxic hemiparesis, sensorimotor strokes, and dysarthria-clumsy hand syndrome. A key indicator of hemorrhagic CSVD is cerebral microbleeds, which clinically resemble lacunar infarctions (10). The pathological basis of cognitive dysfunction may relate to theories involving long-association fiber damage and disruption of the fronto-subcortical circuit. Previous research has shown that CSVD patients exhibit a substantial decline in brain information processing speed, which is associated with damage to white matter fiber structure injury (11). Additionally, CSVD lesions typically damage the cortical and subcortical pathways, with the size, location, and collateral circulation of the infarcts significantly impacting cognitive dysfunction (12). Moreover, a study carried out by Verdelho et al. concluded that the severity of white matter lesions is a predictor of cognitive dysfunction and dementia (13). Furthermore, cognitive decline is associated with the number and location of cerebral microbleeds observed on SWI imaging. Lastly, the impact of CSVD on cognitive function might also include delayed memory, language fluency, information processing capacity, and attention (14).

CSVD can also result in geriatric depression, abnormal gait and posture, and urinary dysfunction. Multiple cross-sectional studies determined that CSVD is a risk factor for gait and postural abnormalities, with the severity of CSVD correlating with the risk of falls and balance disorders (15). Prior investigations have observed that patients with CSVD exhibit symptoms of urinary dysfunction early in the disease course, preceding the decline in cognitive function and gait disturbances (16). A large cohort study on white matter lesions demonstrated that over 70% of patients with white matter lesions (WML) exhibit clinical manifestations of urinary dysfunction, including increased nocturia, frequency, and a sensation of incomplete emptying (17). An increasing number of studies suggest that CSVD is an independent risk factor for psychiatric disorders in middle-aged and older adults. The underlying mechanisms might involve alterations in the non-motor pathways beneath the frontal lobe facilitated by deep penetrating small artery infarction or chronic ischemia/hypoxia, leading to depression.

### Neuroimaging features of cerebral small vessel disease

**Figure 3:**
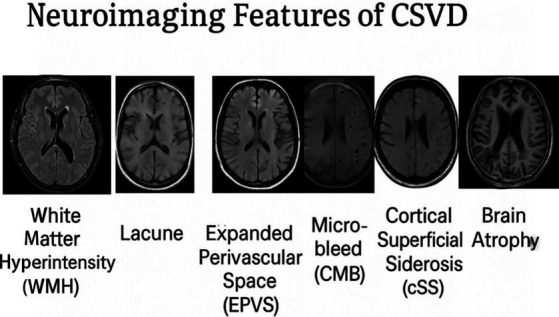
Representative MRI findings in cerebral small vessel disease. Schematic representations of the four key MRI findings in CSVD. White Matter Hyperintensities (WMH): T2-FLAIR sequences showing hyperintense lesions in periventricular and deep white matter. Enlarged Perivascular Spaces (EPVS): T2-weighted images showing small, round/linear hyperintensities following the course of penetrating vessels. Cerebral Microbleeds (CMBs): SWI/T2 sequences showing small, round hypointense lesions representing hemosiderin deposits. Lacunar Infarctions (LI): Appearing hypointense on T1 and hyperintense on T2/FLAIR as fluid-filled cavities (3-15 mm) in deep brain structures*.

Cerebral Small Vessel Disease (CSVD) typically manifests in neuroimaging as enlarged perivascular spaces (EPVS), cerebral microbleeds (CMBs), white matter lesions (WML), lacunar infarctions (LI), and brain atrophy (MTLA). Of note, these features can exist independently or concurrently (see Figure 3).

#### Neuroimaging characteristics of EPVS

Perivascular spaces (PVS) are normal anatomical structures within the nervous system, formed by the extension of the pia mater along penetrating arteries and draining veins into the brain parenchyma, playing a pivotal role in interstitial fluid drainage and immune function. On the other hand, EPVS refers to perivascular spaces larger than 2 mm, typically appearing circular or elliptical with smooth edges. On cranial MRI, EPVS displays signals similar to cerebrospinal fluid on T2-weighted and FLAIR sequences, currently without mass effect or enhancement (18). Recent advances in EPVS research have transformed our understanding of their role in CSVD and cognitive function. While previously considered benign or incidental findings, contemporary studies have established EPVS as important biomarkers of small vessel pathology and predictors of cognitive decline.

Ballerini et al. (19) conducted a longitudinal study demonstrating that baseline EPVS burden in the basal ganglia independently predicts future cognitive decline over a 4-year follow-up period, even after adjusting for other CSVD markers. The differential impact of EPVS location has been clarified by Dumitriu et al. (20), who found that basal ganglia EPVS correlate strongly with executive dysfunction and processing speed impairment, while centrum semiovale EPVS associate more closely with memory deficits.

Quantitative EPVS assessment techniques have advanced significantly, moving beyond visual rating scales to automated detection methods. Sudre et al. (21) developed a machine learning algorithm that demonstrates 94% accuracy in EPVS detection and quantification, allowing more precise evaluation of the relationship between EPVS burden and cognitive function.

Furthermore, the relationship between EPVS and other CSVD markers has been elucidated by Zhang et al. (22), who found that increased EPVS count correlates with WMH progression (r = 0.67, *p* < 0.001) and predicts new lacunar infarct formation, suggesting that EPVS may serve as an early marker of CSVD progression before other radiological features become apparent.

EPVS are now recognized as part of the glymphatic system, playing a crucial role in brain waste clearance. Dysfunction of this system, as evidenced by increased EPVS, may contribute to accumulation of neurotoxic waste products and subsequent neurodegeneration. This represents a novel mechanism linking EPVS to cognitive impairment beyond traditional vascular pathways (23). Brain parenchymal damage caused by EPVS is rarely reported and is generally considered benign or a normal variant that has not yet received substantial attention.

#### Neuroimaging characteristics of CMBs

CMBs are small, acute, chronic, or subacute punctate hemorrhages detectable by MRI sensitive to iron deposition that are characterized by hemosiderin deposits surrounding small cerebral vessels. In SWI sequences, they appear as low-signal lesions, predominantly distributed in the cortex and subcortex, followed by the basal ganglia and thalamus, and less frequently in the cerebellum. Recent research has significantly advanced our understanding of the relationship between CMBs and cognitive function beyond the simple correlation of CMB presence with general cognitive decline.

Differentiation by CMB location has emerged as critical for understanding specific cognitive effects. Werring et al. (24) demonstrated that lobar CMBs (suggesting cerebral amyloid angiopathy) are specifically associated with memory and visuospatial deficits, while deep CMBs (indicating hypertensive arteriopathy) correlate more strongly with executive dysfunction and processing speed impairment. This topographical specificity helps explain the heterogeneous cognitive profiles observed in CSVD patients.

The concept of CMB threshold effects has been established by longitudinal studies. Li et al. (25) identified that the presence of ≥3 CMBs increases the risk of cognitive decline by 2.7-fold (95% CI 1.8–4.2) over a 5-year period, with an accelerated decline observed in patients with ≥5 CMBs. This dose–response relationship provides clinically useful risk stratification markers.

Progression rates of CMBs have also been linked to cognitive outcomes. Wong et al. (26) found that patients with a CMB accumulation rate >2 per year showed a significantly faster decline in global cognition (*β* = −0.31, *p* = 0.003) and executive function (*β* = −0.42, *p* < 0.001) compared to those with stable CMB counts.

The interaction between CMBs and other CSVD markers has been clarified by recent multicenter studies. The UVCI study (27) demonstrated synergistic effects between CMBs and white matter hyperintensities, where the combination of both markers predicted a 3.5-fold greater cognitive decline than either marker alone.

Advanced susceptibility-weighted MRI techniques now allow better characterization of CMB composition and surrounding tissue. Quantitative susceptibility mapping (QSM) studies by Chen et al. (28) revealed that the degree of iron deposition in and around CMBs correlates with the severity of information processing speed deficits, suggesting that iron-mediated neurotoxicity may represent an additional mechanism of cognitive impairment. Studies evinced that the presence of three or more CMBs increases the risk of cognitive dysfunction, particularly affecting the brain’s information processing speed and memory (29).

#### Neuroimaging characteristics of WML

First described by Hachiski et al. (30), WMLs appear on CT images as patchy low-density areas in the centrum semiovale or adjacent to lateral ventricles. With advancements in imaging technology, cranial MRI currently depicts WMLs as multiple punctate high-signal lesions on T2-weighted images, typically perpendicularly aligned with the lateral ventricular walls and symmetrically distributed, also referred to as white matter hyperintensities (WMH). Contemporary research has substantially extended our understanding of WML beyond their visual characterization to include quantitative assessment, progression patterns, microstructural properties, and their relationship to cognitive outcomes.

Quantitative volumetric analysis of WML has largely replaced visual rating scales in research settings. The longitudinal LADIS study (31) demonstrated that annual WMH volume increase of >5% predicts conversion to dementia with 68% sensitivity and 84% specificity. This quantitative approach provides more precise tracking of disease progression than the traditional Fazekas scale.

The heterogeneity of WML has been established by recent diffusion tensor imaging studies. Wardlaw et al. (32) identified distinct microstructural signatures within visually similar WMH, with some regions showing predominantly demyelination patterns while others exhibit axonal loss, suggesting different underlying pathological processes that may respond differently to treatment.

Strategic location of WML has emerged as more important than total volume in determining cognitive outcomes. The brain connectivity hypothesis proposed by ter Telgte et al. (33) demonstrates that WML affecting major white matter tracts (particularly the superior longitudinal fasciculus and cingulum) produce disproportionately severe cognitive effects compared to similar volumes in less strategically connected regions.

WML progression trajectories have been characterized by Sudre et al. (34), who identified three distinct patterns: slow accumulators (27%), steady progressors (45%), and rapid progressors (28%). The rapid progression group showed a 3.8-fold increased risk of converting to dementia within 5 years, independent of baseline WML volume.

The dynamic nature of WML has been revealed by high-field 7 T MRI studies showing that approximately 5–10% of established WMH can partially regress over time, challenging the notion that WML progression is invariably unidirectional and highlighting potential recovery mechanisms that could be therapeutic targets (35). Interestingly, studies employing visual scoring methods such as the Fazekas scale have identified a significant correlation between the progression of WMH and cognitive decline (36).

#### Neuroimaging characteristics of LI

Lacunar infarctions are ischemic infarcts smaller than 20 mm in diameter and are generally caused by occlusion of small penetrating arteries in the cerebral hemispheres or brainstem. On T1-weighted images, LI appears as low or isointense signals; on FLAIR and T2-weighted images, it presents as high signal intensities. Recent advances in LI research have refined our understanding of their formation, evolution, and relationship to cognitive outcomes.

The concept of “lacunar shape signatures” was introduced by Liang et al. (37), who demonstrated that irregular, non-ovoid lacunes are associated with more severe cognitive impairment than regular, ovoid lacunes of similar size, suggesting different pathophysiological mechanisms or varying degrees of tissue repair.

The temporal evolution of acute lacunar infarcts has been characterized using serial MRI by the SILENCE consortium (38). Their work identified three distinct evolution patterns: cavitation (62%), partial regression (26%), and complete disappearance (12%). Importantly, the cavitation pattern was most strongly associated with subsequent cognitive decline, particularly in executive function domains.

Strategic locations for lacunar infarcts have been mapped in relation to cognitive impact. Thalamic lacunes affecting the anterior or dorsomedial nuclei produce disproportionate executive dysfunction due to disruption of frontothalamic circuits, as demonstrated by Zhang et al. (39) using combined structural and functional connectivity analyses.

The phenomenon of “lacunar status” (multiple lacunes in close proximity) has been identified as carrying particularly high risk for cognitive impairment. Chen et al. (40) found that patients with clustered lacunes (≥3 within 5 mm) exhibited 2.4-fold greater cognitive decline over 3 years compared to those with the same number of widely distributed lacunes.

Advanced diffusion imaging techniques have revealed the impact of lacunes beyond their visible boundaries. The concept of the “lacunar penumbra” proposed by Wardlaw et al. (41) describes a 4-8 mm perimeter of tissue surrounding lacunes with altered microstructural integrity that contributes to network disruption and cognitive impairment beyond what would be expected from the visible lesion alone. Common etiologies of LI include diabetes and hypertension, with pathogenesis involving lipohyalinosis, arteriosclerosis, small thrombi occluding deep penetrating arteries, and diabetic microangiopathy (42).

#### Neuroimaging characteristics of MTLA

Brain atrophy is the result of a reduction in brain tissue volume, widened cerebral sulci, and decreased grey or white matter volume caused by various factors, including chronic cerebral ischemia/hypoxia, brain trauma, genetics, encephalitis, alcohol poisoning, and carbon monoxide poisoning.

### Application of functional MRI in cognitive impairment of patients with cerebral small vessel disease

**Figure 4:**
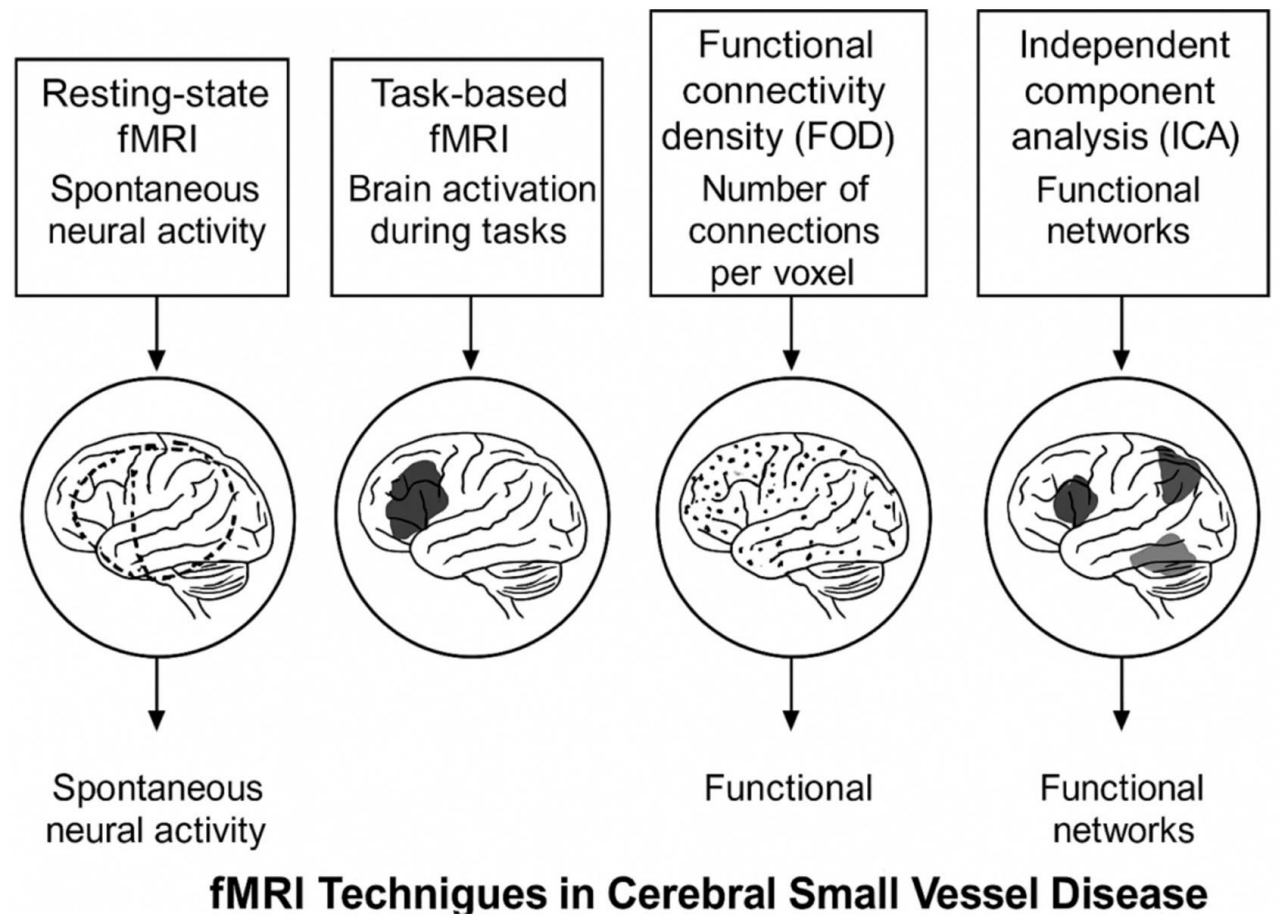
Functional MRI Techniques in CSVD Assessment. Illustration of the four main functional MRI techniques used in CSVD assessment. Diffusion Tensor Imaging (DTI): Tracks water diffusion along white matter tracts to assess microstructural integrity, with key metrics including fractional anisotropy (FA) and mean diffusivity (MD). Resting-State fMRI (rs-fMRI): Measures spontaneous neural activity and functional connectivity between brain regions, identifying network disruption patterns. Magnetic Resonance Spectroscopy (MRS): Quantifies brain metabolites such as N-acetylaspartate (NAA), creatine (Cr), choline (Cho), and myo-inositol (mI) to detect neuronal integrity and membrane turnover. Arterial Spin Labeling (ASL): Non-invasively measures cerebral blood flow and arterial transit time, often revealing the earliest detectable changes in CSVD.

Cerebral small vessel disease (CSVD) clinically manifests as stroke, depression, and cognitive impairment. Structural Magnetic Resonance Imaging (MRI) reveals white matter hyperintensities (WMH), which can be quantitatively measured and may appear earlier in the disease course compared to other biomarkers. Existing research has established that WMH is associated with deficits in information processing and executive function. Recently, rapid advancements in functional MRI technology have broadened its application in medical research and clinical diagnosis, offering greater insights into brain function studies (see Figure 4).

#### Diffusion tensor imaging (DTI)

DTI represents a significant advancement in functional neuroimaging that has transformed our understanding of CSVD-related cognitive impairment. This technique allows for the visualization of white matter microstructure and connectivity by measuring the diffusion of water molecules in biological tissues.

##### Fundamental principles of DTI

DTI involves tracking the extent of diffusion of water molecules in various directions using sensitive gradients. In normal white matter, water diffusion is anisotropic (directionally dependent) due to the organized structure of axonal membranes and myelin sheaths, which restrict perpendicular movement. This anisotropic diffusion can be quantified through several parameters, with fractional anisotropy (FA) and mean diffusivity (MD) being the most commonly utilized metrics.

##### Parameter interpretation

- Fractional Anisotropy (FA): A scalar value between 0 and 1 that describes the degree of directionality of water diffusion. Higher FA values indicate greater integrity of white matter fibers.
- Mean Diffusivity (MD): Reflects the average magnitude of water diffusion. Increased MD suggests breakdown of tissue microstructure.
- Axial Diffusivity (AD): Measures diffusion along the principal axis, with decreases potentially indicating axonal damage.
- Radial Diffusivity (RD): Quantifies diffusion perpendicular to the principal axis, with increases suggesting myelin degradation.

DTI abnormalities typically precede conventional MRI findings in CSVD, making this technique uniquely valuable for early detection. A typical manifestation of impaired white matter structural integrity in DTI is a decrease in FA values and an increase in MD values, often observed in normal-appearing white matter on conventional MRI sequences.

DTI technology can aid in visualizing minor damages and categorize the cortex into multiple regions, thus enhancing the correlation between lesion structure and cognitive dysfunction. Indeed, it plays a decisive role in the early assessment of CSVD, detecting ultrastructural damage in healthy areas and evaluating the integrity of the white matter microstructure (43). Clinically, multiple cross-sectional and longitudinal studies have demonstrated a close correlation between clinical deficits and CSVD (44). Cognitive function is contingent upon the effective operation of a distributed brain network connected by white matter fibers. In normal-appearing white matter, the destruction of microstructural integrity in some areas can lead to cognitive dysfunction. DTI studies in patients with leukoaraiosis have concluded that global histograms of MD and FA maps and voxel-based analyses are closely correlated with the degree of motor and cognitive impairments (45). In a follow-up study carried out by Mielke et al. (46), 26% of patients with cognitive dysfunction progressed to Alzheimer’s Disease (AD), exhibiting aberrant changes in DTI values in hippocampal downstream brain regions and the hippocampus during cognitive dysfunction progression. The memory function in specific brain regions of patients differs based on DTI parameters, with higher MD values in the hippocampus indicating a higher likelihood of microstructural changes in specific brain regions leading to cognitive dysfunction. It is worthwhile emphasizing that cross-sectional studies (47) have demonstrated that DTI is superior to conventional MRI markers such as WMH volume, hippocampal volume, and the number of lacunar infarcts. A novel DTI analytical approach adopted the concept of the brain as a network of cortical nodes, wherein graph theory derived mathematical measures of brain connectivity. Utilizing this method, cortical segmentation into regions is performed using automated software based on anatomical boundaries, with DTI processing methods determining the strength of DTI connections between each node. Indeed, using DTI technology to analyze the imaging representation of SCVD patients can predict future disease progression.

##### Clinical applications of DTI in CSVD

1. Early Detection and Monitoring: DTI can identify microstructural damage in normal-appearing white matter before visible lesions develop on conventional MRI. Lawrence et al. (48) demonstrated that DTI abnormalities in normal-appearing white matter predict development of WMH with 78% accuracy over a 3-year follow-up period.
2. Cognitive Domain Specificity: Recent studies have linked specific DTI metrics with particular cognitive domains. Tuladhar et al. (49) found that reduced FA in the anterior thalamic radiation and forceps minor specifically correlates with executive dysfunction (r = 0.67, *p* < 0.001), while reduced FA in the uncinate fasciculus and cingulum correlates with memory impairment (r = 0.59, *p* < 0.01).
3. Predictive Biomarker: DTI parameters have emerged as powerful predictors of disease progression. In a landmark study by Wardlaw et al. (50), baseline MD values in normal-appearing white matter predicted conversion to vascular cognitive impairment with greater accuracy (AUC = 0.82) than conventional MRI markers including WMH volume (AUC = 0.68) and lacune count (AUC = 0.64).
4. Treatment Response Monitoring: DTI metrics are increasingly used as surrogate endpoints in clinical trials. The PRESERVE DTI study (51) demonstrated that aggressive blood pressure control resulted in stabilization of FA values in strategic white matter tracts compared to standard treatment, correlating with preserved cognitive function despite similar effects on conventional MRI markers.

#### Resting-state functional magnetic resonance imaging (rs-fMRI)

rs-fMRI has revolutionized our ability to examine functional brain networks and their disruption in CSVD. This technique allows for the assessment of functional connectivity patterns while patients are at rest, eliminating task-related confounds and making it particularly valuable for studying patients with cognitive impairment who may struggle with task performance.

##### Fundamental principles of rs-fMRI

rs-fMRI reflects spontaneous neuronal activity based on fluctuations in blood oxygen level signals in a resting state. It relies on the principle that alterations in cerebral blood flow and local oxygen consumption change the local magnetic field, producing detectable blood oxygen level-dependent (BOLD) signals. These signals exhibit low-frequency fluctuations (LFFs) that reflect spontaneous neural activity. Brain regions with similar functions show temporal synchronization of these fluctuations, forming identifiable functional networks even in the absence of external tasks.

##### Analytical approaches in rs-fMRI

1. Functional Connectivity Analysis: Examines temporal correlations between BOLD signals from different brain regions
2. Independent Component Analysis (ICA): Identifies spatially independent networks without a priori assumptions
3. Amplitude of Low-Frequency Fluctuation (ALFF): Measures the intensity of spontaneous brain activity
4. Regional Homogeneity (ReHo): Evaluates local synchronization of spontaneous brain activity
5. Graph Theory: Analyzes network properties including efficiency, clustering, and centrality
6. Dynamic Functional Connectivity: Captures temporal variations in functional connectivity patterns

rs-fMRI reflects spontaneous neuronal activity based on fluctuations in blood oxygen level signals in a resting state. It is uniquely valuable for detecting functional activity changes across the whole brain or specific brain areas. This technique relies on the principle that alterations in cerebral blood flow and local oxygen consumption alter the local magnetic field. Spontaneous blood oxygen level-dependent (BOLD) signals exhibit low-frequency fluctuations (LFFs), which can stimulate spontaneous neural activity. Each brain region exhibits these low-frequency waves, forming a spatial structure similar in function, termed functional connectivity. This approach is extensively applied to identify a range of functional brain networks. Commonly employed methods for analyzing resting-state fMRI data include independent component analysis (ICA), graph theory, amplitude of low-frequency fluctuation (ALFF), and regional homogeneity (ReHo), assessing neuronal function through contrast-enhanced principles.

ICA is an early technique for extracting identifiable networks for analysis, assessing the correlation and association strength of the whole brain’s other voxels with seed points. A decline in functional connectivity may underlie cognitive impairment. As early as 2014, Yao et al. (52) observed that the decrease in functional connectivity primarily occurred between the amygdala, temporo-occipital systems, and the Default Mode Network (DMN). At the same time, Song et al. (53) analyzed the significance of the Salience Network (SN), which involves shifting between affective, attentional, emotional, and cognitive resources, and pointed out that it significantly affected patients with mild cognitive impairment. SN-specific functional changes and interactions with other networks may be potential mechanisms of loss in these individuals. Graph theory analyzes brain lesions from a holistic perspective. Using this approach, Qin et al. (54) studied the relationship between thalamic lacunes and brain network topology and claimed that CSVD patients with thalamic lacunes had reduced overall efficiency and increased characteristic path length, showcasing the pivotal role of thalamic lacunes in mediating neurological function in CSVD patients with mild cognitive impairment. Commonly employed functional segregation methods include ALFF and ReHo. Gao et al. (55) used ALFF analysis to correlate with cognitive function impairment, discovering a close relationship between the frontal lobe and cognitive function. Decreases in ALFF values in certain frontal regions, particularly the orbicular section of the middle frontal gyrus and the left orbital part of the superior frontal gyrus, correlated with cognitive decline. Another study indicated that dynamic ALFF variability in left middle temporal gyrus abnormalities was significantly correlated with cognitive performance in CSVD patients (56). Hence, a reduction in certain regions of ALFF values leads to declines in executive and attentional cognitive functions. ReHo measures the consistency of time signals between a voxel and its surrounding voxels. In other words, a higher ReHo value reflects higher consistency among local voxels but does not necessarily signify increased spontaneous neural activity in the local brain area. In line with the observations of Chen et al. (57), reduced ReHo values in the left temporal lobe, occipital lobe, and left middle temporal gyrus are indicative of lower spontaneous brain activity in these areas, partially reflecting overall cognitive impairment. The executive and attentional impairments in CSVD are reliant on multiple interconnected and interacting networks, mainly including the frontoparietal control network (FPCN), DMN, and Dorsal Attention Network (DAN). Liu et al. (58) implied that CSVD influences the connectivity of the FDCN, with changes in FDCN connectivity related to cognitive changes, suggesting that FDCN may compensate for network damage in CSVD patients with cognitive impairment.

##### Key network findings in CSVD

1. Default Mode Network (DMN): The DMN, active during rest and internal thought processes, shows consistent disruption in CSVD. Recent work by Zhang et al. (59) revealed that DMN connectivity reduction correlates with global cognitive performance (r = 0.64, *p* < 0.001) and specifically with episodic memory (r = 0.71, *p* < 0.001).
2. Frontoparietal Control Network (FPCN): This network, critical for executive control and working memory, shows altered connectivity patterns in CSVD. Wang et al. (60) demonstrated that FPCN hyperconnectivity in early CSVD may represent compensatory mechanisms that eventually fail as disease progresses.
3. Network Integration: Rather than considering networks in isolation, contemporary research emphasizes network integration. The triple-network model (DMN, SN, and Central Executive Network) proposed by Menon (61) for CSVD suggests that cognitive impairment results from dysregulation in the dynamic interplay between these networks rather than dysfunction of any single network.
4. Dynamic Functional Connectivity: Moving beyond static connectivity measures, dynamic functional connectivity analysis has revealed increased temporal variability in network connections in CSVD patients. Li et al. (62) found that greater instability in DMN-FPCN connectivity predicted poorer executive function, independent of structural damage.
5. Structure–Function Relationships: Advanced multimodal analyses have established relationships between structural and functional changes. Chen et al. (63) demonstrated that white matter hyperintensities strategically located at network nodes have greater impact on functional connectivity than those affecting connection edges.

It is important to note that while multiple brain regions show abnormal functional changes in CSVD, these do not necessarily indicate damage to all these areas simultaneously in individual patients. Rather, they reflect the distributed nature of brain networks and the propagation of dysfunction through connected systems. The specific pattern of network disruption varies between patients and correlates with their individual cognitive profiles.

#### Magnetic resonance spectroscopy (MRS)

MRS provides unique insights into the biochemical and metabolic alterations in CSVD that complement structural and functional connectivity analyses. This technique allows for non-invasive measurement of brain metabolites that serve as markers of neuronal integrity, membrane turnover, glial function, and energy metabolism.

##### Fundamental principles of MRS

MRS imaging technology is based on the principle of chemical shift caused by differences in the precession frequencies of identical atomic nuclei due to differing molecular structures. This allows for the differentiation of various compounds based on frequency variations. In brain tissue studies, proton MRS (1H-MRS) is predominantly used clinically, with its spectral peaks reflecting specific metabolite concentrations.

##### Key metabolites in CSVD assessment

1. N-acetylaspartate (NAA): A neuronal marker with peak at 2.0 ppm, synthesized in mitochondria and found primarily in neurons and axons. Decreased NAA indicates neuronal damage or loss.
2. Choline (Cho): Reflects membrane turnover with peak at 3.2 ppm. Increased Cho often indicates accelerated membrane turnover in processes such as demyelination.
3. Creatine (Cr): Present in both neurons and glial cells with peak at 3.0 ppm. Relatively stable in concentration, often used as a reference.
4. Myo-inositol (MI): A glial marker with peak at 3.5 ppm. Increased MI often indicates gliosis or microglial activation.
5. Glutamate and Glutamine complex (Glx): Excitatory neurotransmitters and precursors, with peaks between 2.1–2.4 ppm.
6. Lactate: Marker of anaerobic metabolism, appears as a doublet at 1.3 ppm. Elevated in acute ischemia.

##### Metabolite ratios

The NAA/Cr ratio and Cho/Cr ratio are commonly used to normalize measurements and evaluate neuronal function and membrane integrity, respectively.

MRS imaging technology is based on the principle of chemical shift caused by differences in the precession frequencies of identical atomic nuclei due to differing molecular structures. This fundamental aspect of MRS allows for the differentiation of various compounds based on frequency variations. Currently, 1H-MRS is widely used in clinical settings, with its metabolic products reflecting changes in the metabolism of living tissues. In brain tissue studies using 1H-MRS, commonly measured metabolites include choline (Cho), N-acetyl aspartate (NAA), creatine (Cr), myo-inositol (MI), glutamine and glutamate complex (Glx), and the NAA/Cr ratio.

In 1H-MRS studies of brain tissue, the peak of the Cho wave is observed at 3.2 ppm. An increase in MRS peak values frequently indicates an acceleration in the metabolic renewal of cell membranes of tumor cells, typically involving the precursor of acetylcholine-choline. Studies have shown that an increase in Cho is related to acute demyelination, suggesting a positive correlation between Cho elevation and ischemic white matter loosening (64). The rise in Cho might be ascribed to the breakdown of neuronal cell membranes and neuro-lipids. The peak frequency of the NAA wave, a metabolite synthesized within mitochondria and primarily found in neurons and axons, representing neuronal vitality, is at 2.0 ppm. A decrease in NAA is reflective of neuronal damage and loss. The peak of the Cr wave is at 3.0 ppm, uniformly distributed in brain tissue and present in neurons and glial cells. The level of Creatine (Cr) in brain tissue is relatively stable, serving as an internal reference standard (65). However, in certain pathological conditions such as severe metabolic disorders or extensive tissue damage, Cr levels may also be affected.

Conversely, a decrease in Cr suggests a reduction in the number of neurons, especially astrocytes, at the CSVD lesion site. The NAA/Cr and Cho/Cr ratios are commonly used to evaluate neuronal function and the integrity of the neural myelin sheath. The peak of the MI wave, a product of hormone-sensitive neural receptors that are mainly involved in regulating osmotic pressure, nourishing cells, antioxidation, and synthesizing cell surface-active substances, is situated at 3.5 ppm. The NAA/Cr and Cho/Cr ratios are indicators of neuronal function, assessing the extent of damage and integrity of neurons and myelin. A decrease in the NAA/Cr ratio is correlated with pathological changes such as neural fiber degeneration, swelling, and axonal loss (65). Shukla et al. (66) described that cognitive impairments in Parkinson’s disease patients are associated with decreases in NAA/Cr and NAA/Cho values. Similarly, Harshfield et al. (67) reported that in CSVD patients, a decrease in NAA and an increase in MD in the centrum semiovale white matter suggest a link between cognitive impairments, metabolic abnormalities, and structural damage in this white matter region. Studies indicate (68) that chronic underperfusion leading to CSVD brain white matter damage drives the death of oligodendrocytes and neurons, as well as neural fiber demyelination. Applying MRS technology can aid in identifying pathological metabolic conditions in CSVD lesion areas, providing insights into abnormal neurotransmitter transmission and the destruction of neuronal cells and axons in the brain, as well as into reliable imaging indicators for further elucidating the neuropathological mechanisms underlying CSVD.

##### Recent advances in MRS applications for CSVD

1. Regional Metabolic Profiles: Huang et al. (69) identified specific metabolic signatures in different brain regions affected by CSVD. Normal-appearing white matter adjacent to WMH shows decreased NAA/Cr ratios before visible lesions appear, suggesting metabolic changes precede structural abnormalities.
2. Longitudinal Metabolic Changes: Chen et al. (70) conducted a 3-year longitudinal study demonstrating that the rate of NAA decline in normal-appearing white matter predicts both WMH progression and cognitive deterioration with greater sensitivity than structural measurements alone.
3. Correlation with Cognitive Domains: Recent research by Wang et al. (71) established specific correlations between metabolite ratios and cognitive domains: NAA/Cr ratios in frontal white matter correlate strongly with executive function (r = 0.72, *p* < 0.001), while temporal lobe NAA/Cr correlates with memory performance (r = 0.68, p < 0.001).
4. Metabolic Network Analysis: Moving beyond single-voxel analyses, multivoxel spectroscopic imaging now allows for “metabolic connectivity” analyses. Zhang et al. (72) demonstrated that disrupted metabolic networks (particularly NAA correlations across regions) predict cognitive impairment better than isolated regional measurements.
5. Treatment Monitoring: MRS has emerged as a sensitive tool for monitoring therapeutic interventions. The SPECTRUM trial (73) showed that successful blood pressure control resulted in stabilization of NAA/Cr ratios in patients with hypertension-related CSVD, while these ratios continued to decline in poorly controlled patients despite similar structural MRI appearances.

#### Arterial spin labeling (ASL)

ASL provides critical insights into the hemodynamic abnormalities that precede and accompany CSVD, directly addressing the vascular origins of this disease. As a non-invasive technique for measuring cerebral blood flow (CBF) without contrast agents, ASL has become increasingly important in understanding the relationship between perfusion deficits and cognitive impairment in CSVD.

##### Fundamental principles of ASL

ASL utilizes radiofrequency pulses to tag water molecules in arterial blood, which serve as endogenous tracers. These water molecules freely cross the blood–brain barrier and perfuse brain tissue. By acquiring paired images—one with tagged blood and one without (reference)—and subtracting them, a perfusion-weighted image is generated that quantifies regional cerebral blood flow in ml/100 g/min.

##### Technical advances in ASL

1. Pseudo-continuous ASL (pCASL): Now the consensus recommended implementation, providing higher signal-to-noise ratio.
2. Multi-delay ASL: Accounts for arterial transit time variations, particularly important in CSVD.
3. 3D acquisition strategies: Improved spatial coverage and reduced susceptibility artifacts.
4. Background suppression techniques: Enhanced sensitivity to perfusion changes.

Arterial Spin Labeling (ASL) utilizes radiofrequency pulses to tag water molecules in arterial blood, acting as endogenous tracers. These water molecules, minimally affected by the blood–brain barrier, freely penetrate this barrier. In ASL, cerebral blood flow from the same brain layer is collected as a reference image. This process generates a pair of images: one tagged and one reference. The difference in blood flow signals, post-subtraction between tagged and reference images, generates cerebral blood flow maps, thereby reflecting microcirculatory perfusion and vascular distribution in ischemic brain tissues. The random diffusion of endogenous tracers effectively mirrors tissue perfusion levels while avoiding potential renal toxicity and other adverse reactions associated with exogenous contrast agents. Overall, ASL is a safe, non-invasive, convenient, and direct examination technique, particularly sensitive and specific to changes in cerebral ischemia, playing a significant role in early disease diagnosis and prognosis assessment (74). Cerebral blood flow (CBF) is a primary quantitative indicator in ASL.

Furthermore, changes in perfusion might precede alterations in structure, function, and molecular biomarkers. Recent studies have established that changes in regional perfusion in elderly individuals with brain atrophy or mild cognitive impairment are closely correlated with cerebral perfusion and cognitive status. Lu et al. (75) discovered reduced CBF values in the right frontal lobe and bilateral parietal white matter areas in CSVD patients, corroborating that vascular pathology due to prolonged ischemia and hypoxia is widespread. Promjunyakul et al. (76) found that the penumbral areas of WML blood flow determined by ASL are larger than the structural penumbras measured by DTI, suggesting that changes in blood flow precede white matter damage. Research by Camargo et al. (77) showed significantly lower cerebral blood flow in the prefrontal, parietal, and occipital lobes in patients with cognitive impairments, indicating abnormalities in cerebral blood supply. Zhang et al. (78) observed that in CSVD patients, the cerebral white matter blood flow in areas such as the left centrum semiovale and periventricular white matter adjacent to the lateral ventricles was lower than in the control group, with a significant decrease in those with cognitive impairments. This finding suggests that local perfusion insufficiency leading to white matter lesions eventually facilitates the development of cognitive disorders. Besides, studies indicate that cognitive impairments in CSVD patients are closely related to ischemic, low-perfusion injuries (79). This phenomenon could be explained by the reduction in cerebral blood flow perfusion, further damaging white matter nerve fibers, whilst prolonged ischemia and hypoxia due to reduced perfusion may induce gliosis and neuronal apoptosis, further exacerbating damage to white matter nerve fibers. Han et al. (80) used a combination of ASL and T1WI volumetric analysis to further refine brain regions in 26 patients with mild to severe white matter lesions and identified a negative correlation between the severity of white matter hyperintensity lesions and blood flow in those regions. Wang et al. (81) conducted 3D pCASL measurements on 73 participants, divided into a hypertension group of 41 patients and an age-matched control group comprising 32 patients. Compared to the control group, the hypertension group displayed reduced CBF in the whole brain’s gray matter and normal white matter areas. The decrease in CBF values in these areas might elevate the risk of CSVD, suggesting that ischemic mechanisms potentially contribute to the development of CSVD.

##### Key findings in CSVD from ASL studies

1. Spatial Pattern of Hypoperfusion: Contemporary high-resolution ASL studies have mapped the spatial distribution of hypoperfusion in CSVD. Wu et al. (82) demonstrated a characteristic pattern affecting periventricular white matter, deep frontal regions, and watershed territories, consistent with the vulnerability of end-arterial zones.
2. Temporal Dynamics of Perfusion Changes: Longitudinal studies by Chen et al. (83) have established that CBF reduction precedes structural damage by 1–3 years, with areas showing >20% CBF reduction having a 75% probability of developing WMH within 2 years.
3. Transit Time Abnormalities: Beyond simple CBF reduction, multi-delay ASL studies have revealed prolonged arterial transit time (ATT) in CSVD. Zhang et al. (84) found that ATT prolongation in normal-appearing white matter independently predicts cognitive decline and may reflect microvascular dysfunction before detectable hypoperfusion.
4. Cerebrovascular Reactivity: ASL combined with hypercapnic or pharmacological challenges has enabled assessment of cerebrovascular reactivity (CVR). Cheng et al. (85) demonstrated impaired CVR in CSVD patients, particularly in regions surrounding WMH, suggesting compromised vascular reserve that limits adaptive responses to changing metabolic demands.
5. Network-Based Perfusion Analysis: Moving beyond regional analyses, Li et al. (86) introduced “perfusion covariance networks” that examine the coordination of blood flow across brain regions. CSVD patients show disrupted perfusion networks that correlate with cognitive performance independently of structural damage.

### Multimodal integration approaches in CSVD research

Contemporary CSVD research has increasingly moved toward multimodal integration approaches that combine different MRI techniques to provide complementary information about disease mechanisms and progression. These approaches overcome the limitations of single-modality investigations and offer a more comprehensive understanding of the complex relationships between structural damage, functional alterations, metabolic changes, and perfusion deficits in CSVD.

#### Structure–function coupling

The integration of structural (DTI) and functional (rs-fMRI) techniques has revealed important insights into how white matter damage affects brain network function. Zhao et al. (87) demonstrated that the relationship between structural and functional connectivity is significantly altered in CSVD, with evidence of both compensatory hyperconnectivity and disconnection syndromes depending on the disease stage and network involved. Using graph theoretical approaches applied to both structural and functional networks, Wang et al. (88) identified a “connectivity dissociation index” that quantifies the mismatch between structural and functional connectivity patterns, which correlates strongly with cognitive impairment (r = 0.76, *p* < 0.001).

#### Neurovascular coupling

The integration of hemodynamic (ASL) and neural activity (BOLD) measurements has advanced our understanding of neurovascular coupling in CSVD. Chen et al. (89) combined ASL and rs-fMRI to demonstrate that impaired neurovascular coupling—the relationship between neural activity and blood flow response—contributes to cognitive dysfunction independently of absolute perfusion deficits. In particular, regions with preserved neural activity but reduced perfusion (“neurovascular uncoupling”) show accelerated progression to tissue damage within 18 months (90).

#### Microstructural-metabolic relationships

The combination of DTI and MRS has elucidated the relationships between microstructural damage and metabolic alterations. Zhang et al. (91) found that reduced FA values correlate strongly with decreased NAA/Cr ratios in normal-appearing white matter (r = 0.72, *p* < 0.001), suggesting concurrent demyelination and neuronal dysfunction before visible lesions appear. Importantly, regions with metabolic abnormalities but preserved microstructure show high likelihood of future DTI abnormalities, establishing a temporal sequence of pathological changes (92).

#### Multiparametric prediction models

The most advanced approaches integrate metrics from multiple modalities to improve diagnostic accuracy and prognostic value. The CONNECT-VC consortium (93) developed a machine learning model incorporating 24 parameters across DTI, rs-fMRI, ASL, and MRS that predicted cognitive decline with 83% accuracy over a 3-year period, significantly outperforming models based on any single modality or conventional MRI markers. Similarly, Liu et al. (58) demonstrated that multimodal integration improved the classification of CSVD severity and cognitive status, with different modalities contributing differentially to various cognitive domains.

#### Longitudinal multimodal designs

Recent years have seen the implementation of comprehensive longitudinal studies using multiple MRI modalities to track disease progression. The SPECTRUM-CSVD study (94–97) employed annual assessments with DTI, rs-fMRI, ASL, and MRS over 5 years, establishing the temporal sequence of abnormalities: perfusion deficits (ASL) appeared first, followed by metabolic changes (MRS), then microstructural damage (DTI), and finally functional connectivity alterations (rs-fMRI). This temporal sequence provides crucial insights for developing stage-specific interventions and monitoring strategies (see Table 1).

**Table 1:** Comparative summary of functional MRI techniques in CSVD.

Technique	Key metrics	Primary applications	Temporal sensitivity	Key findings in CSVD	Clinical implications
DTI	FA, MD, AD, RD	Microstructural integrity	Precedes visible lesions by 1–2 years	− Decreased FA in NAWM − Increased MD predicts WMH − −Tract-specific changes	Early detection biomarker; Superior to conventional MRI for prediction
rs-fMRI	Connectivity, ALFF, ReHo	Network function	Concurrent with structural damage	− DMN disruption − FPCN compensation − Dynamic connectivity changes	Network-specific interventions; Functional reserve assessment
MRS	NAA/Cr, Cho/Cr, MI	Metabolic status	Precedes structural changes by 6–12 months	− Decreased NAA in NAWM − Increased MI (gliosis) − Region-specific patterns	Metabolic targets for therapy; Treatment monitoring
ASL	CBF, ATT, CVR	Perfusion status	Earliest detectable change (2–3 years)	− Regional hypoperfusion − Prolonged ATT − Impaired CVR	Vascular intervention timing; Risk stratification

FA, Fractional Anisotropy; MD, Mean Diffusivity; AD, Axial Diffusivity; RD, Radial Diffusivity; NAWM, Normal-Appearing White Matter; WMH, White Matter Hyperintensities; ALFF, Amplitude of Low-Frequency Fluctuation; ReHo, Regional Homogeneity; DMN, Default Mode Network; FPCN, Frontoparietal Control Network; NAA, N-acetylaspartate; Cr, Creatine; Cho, Choline; MI, Myo-inositol; CBF, Cerebral Blood Flow; ATT, Arterial Transit Time; CVR, Cerebrovascular Reactivity.

### Future directions

The integration of functional MRI techniques in CSVD research has opened numerous avenues for future investigation and clinical application. As we advance our understanding of this complex disease, several key areas warrant focused attention:

1. Artificial intelligence and machine learning applications

The increasing complexity of multimodal imaging data necessitates advanced analytical approaches. Future research should focus on developing AI-driven algorithms that can integrate data from multiple functional MRI modalities to create personalized risk profiles and treatment recommendations. Deep learning approaches could identify subtle patterns across modalities that human analysis might miss, potentially revealing new biomarkers and therapeutic targets.

2. Ultra-high field MRI (7 T and beyond)

The advent of ultra-high field MRI scanners offers unprecedented spatial resolution and sensitivity. Future studies should leverage these capabilities to investigate microstructural changes at the level of individual cortical layers and small vessel walls. This could provide insights into the earliest pathological changes in CSVD and enable detection of reversible damage before permanent tissue loss occurs.

3. Novel imaging biomarkers

Beyond traditional metrics, future research should explore novel biomarkers such as:

- Glymphatic function imaging to assess waste clearance mechanisms
- Oxygen extraction fraction mapping to evaluate tissue metabolic stress
- Quantitative susceptibility mapping for iron and calcium deposition
- Diffusion kurtosis imaging for more detailed tissue microstructure characterization

4. Therapeutic monitoring and precision medicine

Functional MRI techniques should be integrated into clinical trials as surrogate endpoints for therapeutic efficacy. Future studies should establish standardized protocols for multimodal imaging in CSVD, enabling cross-center comparisons and meta-analyses. The development of imaging-based precision medicine approaches could match patients to specific interventions based on their unique imaging profiles.

5. Integration with non-imaging biomarkers

Future research should combine functional MRI findings with blood biomarkers, genetic profiles, and digital health data to create comprehensive disease models. This multi-domain approach could improve risk stratification and enable earlier intervention in at-risk populations.

6. Longitudinal natural history studies

Large-scale, multicenter longitudinal studies using standardized multimodal protocols are needed to establish the natural history of imaging changes in CSVD. These studies should span decades to capture the full spectrum of disease progression from preclinical stages to dementia.

7. Development of imaging-guided interventions

Future work should focus on developing interventions that target specific imaging abnormalities. For example, perfusion-based therapies for regions with reduced CBF, or metabolic support for areas showing decreased NAA levels. Real-time imaging feedback could guide personalized rehabilitation strategies.

8. Standardization and accessibility

To translate research findings into clinical practice, future efforts should focus on:

- Developing automated analysis pipelines accessible to non-specialist centers
- Creating standardized reporting templates for functional MRI in CSVD
- Establishing normative databases across diverse populations
- Reducing scan times while maintaining diagnostic quality

## Summary

This article provides a comprehensive overview of cerebral small vessel disease (CSVD), encompassing its general aspects, clinical manifestations, radiological features, and the application of functional magnetic resonance imaging (MRI) in evaluating cognitive impairment. CSVD has emerged as a significant cause of cognitive dysfunction in the elderly, potentially linked to mechanisms such as endothelial damage and blood–brain barrier disruption. Head MRI can reveal characteristic findings like white matter lesions and cerebral microbleeds. Contrastingly, functional MRI techniques, including diffusion tensor imaging, resting-state functional MRI, magnetic resonance spectroscopy, and arterial spin labeling, allow for the assessment of neuronal function and structural integrity. These techniques can detect early cerebral blood flow and metabolic anomalies and predict changes in cognitive function, thus providing radiological support for the etiology, mechanism, and early diagnosis of CSVD-induced cognitive impairments. Our review makes several novel contributions to the field: (1) it provides a comprehensive integration of four different functional MRI techniques specifically focused on cognitive dysfunction in CSVD patients; (2) it synthesizes the latest findings to provide a holistic understanding of the pathophysiological mechanisms; (3) it highlights the relationships between imaging biomarkers and specific cognitive domains; and (4) it discusses emerging multimodal approaches that overcome limitations of single-modality investigations.

While each functional MRI technique provides valuable insights individually, their integration offers the most comprehensive understanding of CSVD pathophysiology. DTI reveals microstructural damage to white matter tracts before conventional MRI abnormalities appear. rs-fMRI demonstrates functional connectivity disruptions between critical brain networks that correlate with specific cognitive deficits. MRS identifies metabolic alterations reflecting neuronal damage and glial activation that may precede structural changes. ASL quantifies perfusion deficits that often represent the earliest abnormalities in the CSVD cascade.

The future of CSVD research lies in multimodal integration approaches and longitudinal designs that can establish the temporal sequence of pathological changes and identify individuals at highest risk for cognitive decline. Such approaches will be essential for developing early intervention strategies, monitoring disease progression, and assessing treatment efficacy. Ultimately, these advanced imaging techniques promise to transform our approach to CSVD from late detection and symptom management to early identification and targeted prevention. Taken together, this review showcased recent advancements in functional MRI related to CSVD-induced cognitive impairment, thus offering valuable insights for further exploration in this field.

## Data Availability

The original contributions presented in the study are included in the article/supplementary material, further inquiries can be directed to the corresponding author.
